# Prevalence of depression among subjects with and without gestational diabetes mellitus in Bangladesh: a hospital based study

**DOI:** 10.1186/s40200-015-0189-3

**Published:** 2015-07-28

**Authors:** Khurshid Natasha, Akhtar Hussain, A. K. Azad Khan

**Affiliations:** Institute of Health and Society, General Practice and Community Medicine, Section for International Health, Faculty of Medicine, University of Oslo, Oslo, Norway; Diabetic Association of Bangladesh, Dhaka, Bangladesh; Department of Health Promotion and Health Education, Division of Public Health, Bangladesh University of Health Sciences, Dhaka, Bangladesh

**Keywords:** Bangladesh, Depression, Epidemiology, Gestational diabetes mellitus

## Abstract

**Background:**

Data on association between depression and diabetes during the pregnancy period in Asia, specifically in Bangladesh are scarce. The study was designed to measure the prevalence of depression during pregnancy with or without Gestational Diabetes Mellitus (GDM).

**Methods:**

Seven hundred and forty eight pregnant women (382 with GDM, 366 without-GDM) attending at the Bangladesh Institute of Research and Rehabilitation in Diabetes, Endocrine and Metabolic Disorders, participated in the study. Blood glucose was measured following both WHO and ACOG criteria; GDM was diagnosed within 24–28 weeks. Depressive symptoms were assessed following MADRS scale. Semi-structured questionnaire was used to record their socio-demographic status and clinical and family history. Blood pressure, height, weight were also measured.

**Results:**

Overall prevalence of depression was 18.32 %. Depression was higher in GDM subjects (25.92 %) compared to without-GDM subjects (10.38 %) with mean age of of 28.34 and 27.17 years respectively. Prevalence of depression was alarming in both the extreme of age. Dwelling place (*P* < 0.009) and past history of GDM (*P* < 0.018) had strong association with Depression. Higher prevalence of depression was found in Primipara whereas the risk of GDM increased with parity. Other obstetrical factors did not show any significant association with depression and GDM. Income (self and total family), physical exercise, sedentary lifestyle and workload had no significant statistical association with depression or GDM.

**Conclusion:**

Higher rate of depression in pregnancy deserves medical attention especially women diagnosed with GDM. Further studies should estimate adverse pregnancy outcome for untreated depression especially in GDM cases.

## Background

Depression affected approximately 350 million people and counted as one of the leading causes of disability worldwide [1]. Prevalence of depressive disorders in Bangladesh is 4.6 % [2]. Depression is more common in women than in men and is the main cause of disease burden in developed and developing countries for women between the ages of 15 and 44 [3, 4]. Since this includes the childbearing years for women, the risk of depression for women during pregnancy and the postpartum period increases. Although many people believe that women are resistant to become depressed during pregnancy, but at least 20 % of women are depressed during this period [5]. Studies of depression and anxiety show their incidences to be approximately 5 % in non-pregnant women, approximately 8–10 % during pregnancy and about 13 % in the year following delivery [3]. An increase in the percentage of antenatal depression has been reported in women with low social support [6–10], low socioeconomic status [7], lower education levels [7, 10–13], and younger age [7, 11, 13, 14]. Antenatal depression is also associated with experiencing more discomfort from pregnancy-related physical symptoms, increased functional impairment, and greater marital conflict. Additionally, antenatal depression is a strong risk factor for postpartum depression, which is associated with poor maternal-infant bonding and may have adverse effects on infant development. Despite these findings, depressive disorders continue to remain underdetected and undertreated in pregnancy [3]. A recent community-based study found prevalence of depression among 671 mothers during their ante-partum period was 18 %, in two rural sub-districts of Bangladesh [15].

Both depression and diabetes are common in pregnancy and have serious consequences for mother and foetus. Depression occurs in 25 % of persons with type 1 and type 2 diabetes [16]. Whether there is more depression in gestational diabetics than without is unclear [17].

Gestational Diabetes Mellitus (GDM) affects approximately 12 % of women all over the world [18]. The prevalence in Asia ranges from 1 to 3 % (north east of Turkey 1.23 %, [19] Japan 2.9 % [20] and China 2.31 % [21]. The diversity in the prevalence of GDM in different countries are resulted from differences in ethnicity and race of population and also the methods and cut off points which are used on screening and diagnosis [22, 23]. GDM leads to increased incidence of postpartum diabetes in mother and some adverse maternal and foetal ramifications during the pregnancy and the postpartum period [24–28]. It may be possible to prevent many maternal and foetal complications by strategies such as timely screening methods and managing blood glucose in afflicted pregnant women.

A study conducted in New Jersey found, prevalence of depression during pregnancy or postpartum was 15.2 % in subjects with GDM but only 8.5 % in subjects without GDM [29]. Other researchers revealed women with pre-existing diabetes had 54 % higher odds of any antenatal depression compared to those without diabetes [30]. Although the association between depression and diabetes is well established, very few studies have examined the association between these disorders during the pregnancy period [31]. In Bangladesh this data is really scarce we feel. Therefore the purpose of the study was to estimate the prevalence rate of depression during pregnancy with or without GDM and to compare the rates of depression. Further the determinants of depression in pregnancy were assessed.

## Methods

### Study population

This study was conducted from August 2011 to September 2012 at outdoor department of the Bangladesh Institute of Research and Rehabilitation in Diabetes, Endocrine and Metabolic Disorders (BIRDEM). It probably has one of the largest outpatient departments in Asia, enrolling 80–100 new patients with diabetes and 2500–3000 diabetics seeking routine follow-up and specialized care every day. From August 2011 to September 2012 a total of 491 new GDM subjects were registered.

### Pilot study

As we did not have any prevalence rate of depression in pregnancy period and with GDM subjects in Bangladesh a pilot study was done prior to the study between June & July 2011 to calculate the prevalence rate. Fifty GDM subjects and 50 subjects without glucose abnormality (without-GDM) were selected to find out the prevalence of depression among them. It was found that 32 and 19 subjects were depressed among GDM (64 %) and without glucose abnormality (38 %) group respectively.

### Main study

Sample size was calculated {from the formula (z^2^ × pq) ÷ d^2^ (*z* = 1,96 *p* = 0,64 *q* = 1-p *d* = 0,05)} based on the pilot study. 354 for GDM group and 350 for without GDM or control group were taken. For ease of calculation and to avoid wash out 400 newly registered pregnant women with GDM and 400 pregnant without glucose abnormality were invited. At the end a total of 748 subjects (382 with GDM and 366 without GDM) participated in this study.

### Exclusion criteria

Subjects with pregnancy over 28th week, diagnosed diabetes prior to pregnancy, old registered GDM subjects, complications due to medical disorder and subjects unwilling to participate (specially to give contact no) were excluded.

### Data collection

#### Diagnosis of gestational age

The gestational age was determined for all of the women based on the last menstrual period and according to the findings of ultrasonography performed between 8th to 20th weeks of gestation.

#### Diagnosis of GDM

The diagnostic test for GDM was done between 24th and 28th weeks of pregnancy. For screening WHO and American Congress of Obstetricians and Gynaecologists (ACOG) [25] criteria was used. Venous blood samples were collected in the morning after an overnight fast of atleast 8 h and 2 h after administration of 75 gm oral glucose. At least 3 days of unrestricted diet and regular physical activity was ensured. Venous plasma glucose (VPG) was measured by the glucose oxidase method using Dimalesion RxL Max (Siemens Healthcare Diagnostics Ltd., Camberley, UK).

The women were diagnosed as a Case of GDM if : Plasma Glucose found ≥7.0 (WHO) or ≥5.3 mmol/L at Fasting, and ≥8.6 mmol/L at 2 h after 75 gm Glucose intake (ACOG), (which ever detected first).

#### Anthropometric measurements

Measurements of height and weight were taken with light clothes without shoes. For height, the subject stood in erect posture vertically touching the occiput, back, hip and heels on the wall while gazing horizontally in front and keeping the tragus and lateral orbital margin in the same horizontal plane.

#### Blood pressure and diagnosis of hypertension

Prior to blood-pressure (BP) measurement, 10 min rest was assured and using standard cuffs for adults fitted with mercury sphygmomanometer minimized variation in measurement. The pressure was measured on the right arm, placing the stethoscope bell lightly over the brachial artery by auscultatory method. Systolic and diastolic pressures were measured in sitting position not cross legged. Two readings were taken 5 min apart, and the mean of the two was taken as the final blood pressure reading of the individual. Hypertension (HTN) was defined (operational) if systolic blood pressure (SBP) was ≥140 mm Hg and/or diastolic blood pressure (DBP) was ≥90 mm Hg was found in three visits or current treatment with antihypertensive medication [32].

#### Face to face interview

At the first visit, data on sociodemographic status and personal information was collected using a pretested semi structured questionnaire. The questionnaire consisted of sets of closed-ended questions regarding demographic data such as age, educational background, dwelling place, religion, average household income classified relative to the minimum wage, occupational status, history of pregnancy, history of diabetes, mental disorder (depression) and specified drug intake, personal habits and lifestyle, family history of DM, HTN and mental illness. If someone was found depressed an additional open ended question was put to find out the cause.

#### Assessment of depressive symptoms

In 1979, Montgomery and Asberg developed a quantitative tool for depression rating scale with 10 questions [33]. The sum of each item is from 0 to 6 thus total sum of the questionnaire ranges from 0 to 60. Since its development, the Montogomery and Asberg Depression Rating Scale (MADRS) has been widely used all over the world, including Bangladesh [34], Pakistan [35], and Srilanka [36]. The rating is based on a clinical interview moving from broadly phrased questions about symptoms to more detailed ones which allow a precise rating of severity. MADRS scores are categorized into 4 groups, Healthy (0–12), Mild depression (13–19), Moderate depression (20–34) and severe depression (35–60) [33]. The questionnaire was translated into local language ‘Bangla’ [37]. Principal researcher was specially trained to conduct the interview by a psychiatrist in Norway who has extensive experience in the assessing depression score by MADRS [37].

### Ethics

The protocol was approved by the Ethical Review Committee of Diabetic Association of Bangladesh. Verbal consent was received from all subjects. Objectives and the procedure of study were oriented to the subjects, including their right to refuse and withdraw at any stage of the study or to bar their data from analyses. All information and data collected for the study, were deemed confidential. every participant received a hardcopy of their own biochemical results.

### Data analysis and statistical methods

The prevalence rate was determined by simple percentages. Statistical comparisons between different groups were made using chi-square test. The odds ratio (OR) with 95 % confidence interval (CI) for the risk factors was calculated assuming the least level of relevant criteria as a reference value. Logistic regression was performed to adjust for potential confounding factors using SPSS 21 and STATA for all statistical analysis (SPSS Inc., Chicago, IL, USA).

## Results

Overall prevalence of depression among pregnant women was 18.32 %. Among the total depressed subjects (137) only one was found severely depressed, 95 were mildly (12.70 %) and 41 were moderately (5.48 %) depressed. The rate was higher in GDM subjects (25.92 %) than without-GDM subjects (10.38 %) (Table 1).

**Table 1:** Socio-demographic characteristics and depression among study population

Characteristics	Whole Population	Subjects without GDM n. (%)	Subjects with GDM n. (%)	Subjects without Depression n. (%)	Subjects with Depression n. (%)
*n* = 748 (%)		366 (*48.93*)	382 (*51.07*)	611 (*81.68*)	137 (*18.32*)
Age in years	27.77 ± 4.93	27.17 ± 4.39	28.35 ± 5.34	28.07 ± 6.48	27.71 ± 4.51
≤ 17	5 (0.66)		5 (*1.31*)	2 (*0.33*)	3 (*2.2*)
18–25	243 (*32.48*)	141 (*38.5*)	102 (*26.70*)	202(33.06)	41 (29.9)
26–35	469 (*62.701*)	*216 (*59.0*)	*253 (66.23)	384 (62.85)	85 (62.0)
36–45	31 (*4.14*)	9 (*2.5*)	22 (*5.76*)	23.00 (*3.76*)	8.0 (*5.8*)
Education years	12.73 ± 3.81	13.90 ± 3.03	11.62 ± 4.14	12.67 ± 3.8	13.67 ± 3.9
0	6 (0.8021)	1 (.3)	5 (1.31)	5 (.8)	1 (.7)
1–5 years	46 (6.1497)	12 (3.3)	34 (8.90)	38 (6.2)	8 (5.8)
6–12 years	266 (35.561)	97 (26.5)	169 (44.24)	220 (36.0)	46 (33.6)
>13 years	430 (57.487)	256 (69.9)	174 (45.68)	348 (57.0)	*82 (59.9)
Occupation					
Housewives	501 (66.979)	226 (61.7)	275 (72.0)	416 (68.1)	85 (62.0)
Students	82 (10.963)	52 (14.2)	30 (7.9)	66 (10.8)	*16 (11.7)
Labours and Farmers	8 (1.0695)	2 (0.55)	6 (1.57)	7 ()1.15	1 (0.73)
Business and Others	10 (1.34)	5 (1.37)	5 (1.31)	9 (1.47)	1 (0.73)
Service holders	147 (19.652)	81 (22.1)	66 (17.3)	113 (18.5)	*34 (24.8)
Self income in BDT					
<5000	12 (1.60428)	3 (.8)	9 (2.4)	9 (1.5)	3 (2.2)
5001–10,000	21 (2.80749)	7 (1.9)	14 (3.7)	19 (3.1)	2 (1.5)
10,001–15,000	36 (4.81283)	17 (4.6)	19 (5.0)	27 ()4.4	9 (6.6)
15,001–20,000	55 (7.35294)	37 (10.1)	18 (4.7)	45 (7.4)	10 (7.3)
>20,001	43 (5.74866)	24 (6.6)	19 (5.0)	29 (4.7)	14 (10.2)
Dependant	581 (77.6738)	278 (76.0)	303 (79)	482 (78.9)	99 (72.3)
Family income in BDT					
<5000	8 (1.06952)	1 (.3)	7 (1.8)	8 (1.3)	
5001–10,000	45 (6.01604)	4 (1.1)	41 (10.7)	36 (5.9)	9 (6.6)
10,001–15,000	99 (13.2353)	37 (10.1)	62 (16.2)	73 (11.9)	26 (19.0)
15,001–20,000	240 (32.0856)	123 (33.6)	117 (30.6)	201 (32.9)	39 (28.5)
>20,001	356 (47.5936)	201 (54.9)	155 (40.5)	293 (48.0)	63 (46.0)
Dwelling area					
Urban	630 (84.22)	*347 (94.8)	283 (74.1)	212 (74.9)	71 (71.7)
Semiurban or Rural	118 (15.78)	19 (5.201)	99 (25.901)	71 (25.1)	28 (28.3)
Depression					
Mild	95 (12,70)	27(7.401)	*68(17.801)		
Moderate	41(5,48)	11(3.001)	*30(7.901)		
Severe	1(0,13)	0	1(0.301)		
Total	137(18,32)	38(10,38)	*99(25,92)		

1USD =Approximatley 80 Bangladeshi Taka (BDT)

Mean age and education years of the study population were 27.77 ± 4.93 and 12.76 ± 3.86 years respectively. Largely the subjects were housewives (501), dependant (581), living in urban areas (630) and came from a family with income of more than Bangladeshi Taka (BDT) 20,000/- per month (356) (Table 2). Mean BMI of the population was 25.87 ± 3.94 with mean SBP 115.51 ± 9.144 mm of Hg and mean DBP 78.88 ± 10.31 mm of Hg. Majority of the subjects had moderate work load in daily life (374). Primipara (295) had the preponderance in this study*.* Few women had previous history of depression (7.74 %). Among them 1.47 % reported as diagnosed and treated by physician, rest were counted by self reporting.

**Table 2:** Distribution of study population according to obstetric, clinical and family history

Characteristics	Whole Population	Healthy	Only Depression	Only GDM	Depression and GDM
*n* = 748	748	328 (43.85)	38 (5.08)	283 (37.83)	99 (13.24)
Mean Gestational Week	25.50 ± 1.41	25.49 ± 1.36	25.60 ± 1.53	25.39 ± 1.42	25.83 ± 1.51
Parity					
1 N (%)	295 (39.43)	148 (45.12)	*20 (52.63)	93 (32.86)	34 (34.34)
2 N (%)	237 (31.68)	113 (34.45)	12 (31.58)	83 (29.33)	29 (29.29)
≥ 3 N (%)	216 (28.87)	67 (20.43)	6 (15.79)	*107 (37.81)	36 (36.36)
History of D & C					
0 N (%)	559 (74.73)	250 (76.22)	31 (81.58)	204 (72.08)	74 (74.75)
1 N (%)	146 (19.51)	61 (18.60)	6 (15.79)	62 (21.91)	17 (17.17)
≥ 2 N (%)	43 (5.74)	17 (5.20)	1 (2.63)	17 (6.01)	8 (8.10)
History of IUD					
0 N (%)	715 (95.58)	317 (96.65)	37 (97.37)	270 (95.41)	91 (91.92)
1 N (%)	28 (3.74)	11 (3.35)	1 (2.63)	9 (3.18)	7 (7.07)
≥ 2 N (%)	5 (0.66)			4 (1.41)	1 (1.01)
History of Neonatal Death					
0 N (%)	724 (96.79)	321 (97.87)	37 (97.37)	269 (95.05)	97 (97.98)
1 N (%)	23 (3.07)	7 (2.13)	1 (2.63)	13 (4.59)	2 (2.02)
≥ 2 N (%)	1 (0.13)			1 (0.35)	
History of GDM	55 (7.35)			*38 (13.4)	*17 (17.2)
History of Depression					
N (%)	47 (11)	13 (4.0)	4 (10.5)	22 (7.8)	8 (18.2)
Diagnosed N (%)	11 (2.1)	5		5 (1.8)	1 (4.0)
History of Depression Related with Pregnancy Period	22 (2.94)	8 (2.4)	2 (5.3)	9 (3.2)	3 (3.0)
History of Taking Sedative	15 (2)	7 (46..7)	2 (13.03)	3 (20.0)	3 (20.0)
Family History of DM					
One or Both Parents	289 (38.6)	95 (28.96)	*21 (55.26)	*127 (44.88)	*46 (46.46)
Relatives other than parents	64 (8.602)	29 (8.84)	2 (5.26)	25 (8.83)	8 (8.08)
Parents and other relatives	51 (6.801)	15 (4.57)	5 (13.16)	23 (8.13)	8 (8.08)
Family History of HTN					
One or Both Parents	304 (40.6)	125 (38.11)	*24 (63.16)	*119 (42.05)	*36 (36.36)
Relatives other than parents	27 (3.601)	12 (3.66)	1 (2.63)	10 (3.53)	4 (4.04)
Parents and other relatives	23 (3.101)	9 (2.74)	1 (2.63)	10 (3.53)	3 (3.03)
Family History of Depression/MD					
One or Both Parents	13 (1.701)	6 (1.83)	1 (2.63)	5 (1.77)	1 (1.01)
Relatives other than parents	10 (1.301)	6 (1.83)		2 (0.71)	2 (2.02)
*Mean Depressive score	6.42 ± 6.89	4.42 ± 5.89	17.92 ± 4.57	4.89 ± 3.92	18.17 ± 5.22
Blood Glucose					
Mean FBG	5.80 ± 1.63	4.43 ± 0.98	4.31 ± 065	6.59 ± 1.37	6.53 ± 1.51
Mean ABF	8.36 ± 2.74	5.87 ± 1.32	5.47 ± 0.69	9.77 ± 2.36	9.63 ± 2.16
Blood Pressure					
Mean SBP	115.51 ± 9.144	115.63 ± 9.11	112.11 ± 9.70	115.90 ± 9.13	115.30 ± 8.91
Mean DBP	78.88 ± 10.31	80.30 ± 12.89	*78.95 ± 10.22	*77.99 ± 7.30	*76.67 ± 6.92
Hypertension	327 (13)	51 (15.58)	4 (10.056)	32 (11.399)	10 (10.00)
Mean BMI	25.87 ± 3.94	24.76 ± 2.75	27.61 ± 4.13	26.68 ± 4.48	27.71 ± 4.46

Elderly (by mean age 28.07 ± 6.48 years) were prone to be depressed and prevalence of depression was alarming in both the extreme of age (Tables 1 and 3). Surprisingly depression found more in higher educated group (>13 years) and descended accordingly (Tables 1 and 2). Mean DBP found lower in subjects with depression, but there was nothing significant in SBP issue. Women with family history of DM especially of parents were more prone to depression. Housewives seemed to suffer more from both diseases than other groups but not statistically significant. In the ‘service holder’ group prevalence of depression seemed to be highest (23.13 %) then posed the ‘students’ group where 19.51 % subjects were depressed. Among the depressed group though most of the subjects were dependant financially but if looked through occupational assemblage subjects who earn more than 20,000 BDT/month were depressed for the most part (32.56 %). Depression rate was lower in subjects who lead sedentary life and do physical exercise (around 18 %) (Table 4). Prevalence of depression found more in primipara subjects. Neither the previous history of depression nor the other obstetrical factors had any significant relation with present depression.

**Table 3:** Age wise prevalence of depression among GDM and without-GDM subjects

Age in years	Prevalence		
	Without-GDM	GDM	Total
≤17	2	3	60.00 (4.55, 5.0)
18–25	9.22 (0.050,05)	27.45 (6.45, 8.55)	*16,87 (3.58, 4.25)
26–35	10.65 (0.04, 0.04)	24.51 (5.26, 6.76)	*18.12 (4.77, 5.75)
36–45	22.22 (0.30, 0.24)	27.27 (7.01, 9.27)	*25.81 (3.40, 4.43)
Total	10.38 (0.03, 0.03)	25.92 (0.00, 0.00)	*18.32 (4.09, 4.93)

**Table 4:** Prevalence of Depression and GDM among different groups according to life style standard

Characteristics *N* = 748	Subjects with GDM n (% within group)	Subjects with Depression n (% within group)
Occupation		
House wife (*n* = 501)	275 (54.89)	85 (16.97)
Students (*n* = 82)	30 (36.59)	*16 (19.51)
Labours and Farmers (*n* = 8)	6 (75.00)	1 (12.50)
Business and Others (*n* = 10)	5 (50.00)	1 (10.00)
Service holders (*n* = 147)	66 (44.90)	*34 (23.13)
Self income in BDT		
Dependant (*n* = 581)	303 (52.15)	99 (17.04)
< 5000 (*n* = 12)	9 (75.00)	3 (25.00)
5001–10,000 (*n* = 21)	14 (66.67)	2 (9.52)
10,001–15,000 (*n* = 36)	19 (52.78)	9 (25.00)
15,001–20,000 (*n* =55)	18 (32.73)	10 (18.18)
> 20,001 (*n* = 43)	19 (44.19)	*14 (32.56)
Family income		
< 5000 (*n* = 8)	7 (87.50)	
5001–10,000 (*n* = 45)	41 (91.11)	9 (20.00)
10,001–15,000 (*n* = 99)	62 (62.63)	26 (26.26)
15,001–20,000 (*n* = 240)	117 (48.75)	39 (16.25)
> 20,001 (*n* = 356)	155 (43.54)	63 (17.70)
Workload		
Mild-workload (*n* = 355)	176 (49.58)	*75 (21.13)
Moderate-workload (*n* = 374)	190 (50.80)	60 (16.04)
Heavy-workload (*n* = 19)	16 (84.21)	2 (10.53)
SEDENTARY LIFESTYLE		
Yes(*n* = 185)	*31 (62.00)	*9 (18.00)
PHYSICAL EXERCISE		
Yes (*n* = 50)	*101 (54.59)	*33 (17.84)

Chi square test proved that there were significant associations between depression and GDM (*P* < 0.000), depression and dwelling place (*P* < 0.009), depression and past-history of GDM (*P* < 0.018), GDM and past-history of GDM (*P* < 0.000).

Subjects with GDM were with higher mean age (28.35 ± 5.34 years) than without GDM subjects. More than 13 years of education and with total family income of more than 20,000 BDT/month showed highest prevalence rate of GDM. We could add that subjects with parental history of HTN and DM were also prone to GDM. Majority of the subjects who lead a sedentary life were suffering from GDM. Risk of GDM increased with parity whereas no other obstetrical factors showed any significant association with GDM. GDM subjects found to be at 3 to 4 folds risk for depression than without-GDM subjects while age, education, income and parity were adjusted (OR 4.06 for mild and 3.9 for moderate depression, *P* < 0.000). The study could not reveal any stable statistical relationship between income (self and total family) and diseases, but eccentrically who earned least seemed more prone to depression (OR 4.7, *P* < 0.05). Logistic regression revealed that income and parity might have some association with GDM and depression which was not clear that way (Table 5).

**Table 5:** Odds ratio: healthy, depressed, gestational diabetic and both

Characteristics	Healthy and depressed only		Without GDM and GDM	
	Odds Ratio	*P* value	Odds Ratio	*P* value
Age in years				
Up to 25				
26–35	1.297089	0.580	.259092	0.005
36–45	3.991071	0.209	.4416422	0.059
Education				
1–5 years	1.374937	0.794	.4640658	
6–12 years	.7491804	0.582	.4364332	0.538
> 13 years	3.991461	0.328	.2090465	0.489
Dependant	na			
< 5000	*4.748693	0.024	1.654802	0.513
5001–10,000	1.735306	0.403	*2.966219	0.029
10,001–15,000	2.686736	0.150	1.340836	0.434
15,001–20,000			.6526762	0.200
> 20,001	na		.8578098	0.672
<5000			empty	
5001–10,000	2.53038	0.215	empty	0.000
10,001–15,000	2.226809	0.086	7.625614	0.172
15,001–20,000	(omitted)		1.46868	0.698
> 20,001	(omitted)			
Parity				
> 1	0.78	0.53	.9822621	0.934
> 2	0.66	0.399	1.510549	0.090
Depression				
No				
Mild			*3.065062	0.000
Moderate			*3.935684	0.000
Severe			empty	

## Discussion

The primary aim of this study was to assess the prevalence of depression in pregnancy. Result of the study was alarming and similar with another study from Bangladesh [15]. Although the severity of depression was not much towering (69.3 % among the depressive subjects was mildly depressed) in this study.

The next objective was to determine if women with GDM had more depression than women without-GDM. Findings indicated that there was not only a significant difference between the prevalence of depression among women with GDM and without-GDM but also between the mean depression scores (8.33 ± 7.23 in GDM and 4.42 ± 5.89 in without-GDM). The outcome is similar with the study from New Jersy [29]. Similar to our study Mautner et al., reported that women with GDM had higher mean scores (*M* = 7.55) when compared to women without GDM (*M* = 6.41) [38]. The relationship between GDM and depression was statistically very significant.

Finding the associated factors was another aspire of this study. In some studies ‘younger age’, [7, 11, 13, 14] and the ‘third trimester’ [8] have been found to be associated with higher levels of depression among GDM subjects. In the current study, logistic regression did reveal that after controlling for age, education, income and gravid, women with GDM were three to four times more prone to have depression than women without GDM. A similar analysis indicated that women with GDM were 2.3 times more likely to have depression when controlling for age, marital status, income, BMI, and parity, but these findings were not statistically significant [3]. In one study there was a significant difference in depressive scores between women with secondary or lower education and those with diploma or higher education [39]. Our study found subjects with higher education and without physical activity were largely in depression. It is assumed that with ‘higher education’, the ‘occupation’ becomes more desk-oriented and competing intellect, which lessens physical activity and rises apprehension resulting huge risk of depression and GDM. Depression rate also ascends with concern of urban lifestyle and diseases perception. This may be the reason behind the elevated rate of depression in high educated group in our study. It was also interesting that the factors like GDM, age and financial status were significant predictors of depression though income was not established statistically. Some of the reviewed literature had reported that age, [7, 10, 11, 13, 14, 40, 41] marital status, [10, 12, 13, 42] and income [7, 11, 40] have impact on depression. In our study 66.97 % participants were housewives (who do not get any salary from outsource) who tend to suffer more from both diseases but not statistically significant and this may not reflect the actual situation as the other categories of occupation were very narrow. Those women who have‘paid jobs’ must continue their pregnancy bearing responsibility for household work also, thus they might get stressed or depressed more. This may explain the reason behind the high prevalence rate of depression among service holders. Somehow similarly students also get worried to continue their study while they get pregnant. Whilst woman is counted as important financial source for the family, particularly then her ‘self income’ might be measured as a factor for depression especially when the monetary status lowers due to the pregnancy leave. This may explain why risk of depression increased (OR 4.74, *P* < 0.05) in lowest incoming subjects but not in middle or high incoming subjects. Meanwhile, in case of GDM or both diseases, risk increases with middle and moderate income. But this issue was not very clear or significant in all financial strata. More than 50 % among the subjects who lead a sedentary life were suffering from GDM but this did not seem to be a factor for depression (<20 %). One meta-analysis resulted in a relative risk (RR) of GDM of 0.91 (95 % CI 0.57 to 1.44), between subjects with physical activity and without [43].

Larger portion of primipara found depressed whereas multipara subjects were in high-risk of GDM. This findings associate with another study from Jordan [39].

Another important finding was that women with a history of depression were more likely to have GDM. This finding suggests that a history of depression is a risk factor in development of GDM. Same finding was established by the study done by Byrn MA [3]. This study agrees that Familial history of type two diabetes (FHD) represents a pathophysiologically unique risk factor for GDM. The subjects who had family history (especially parental) of diabetes were more prone to GDM and even to Depression. Who knows about the family history become anxious and drags stress also which might result in both the complications. Nothing significant could be sketched out from the clinical findings of the subjects.

The sample size of this study was not so large to compare in more categories and make deeper analysis. Social status and within family relationship are also important factors to assess depressive condition which could not be included in this study. Otherwise this study was a noble attempt to find out the mental health status of pregnant women from Bangladesh.

## Conclusion

Our study confirms that depression is common during pregnancy, and education, financial status and GDM of course are the independent factors associated with this. Additional qualitative investigations are needed to explore the socio-economic factors responsible for this. The prevalence of depression is really alarming in our country and since proved that women with GDM were more likely to be depressed than without, health care providers may want to screen women with GDM more frequently for depression during prenatal care visits. Further researches are recommended to find out the significant factors related with depression. These estimates for GDM and depression may help to formulate new policies to prevent and manage them both.

## Abbreviations

WHO: World Health Organization
ACOG: American Congress of Obstetricians and Gynecologists
DAB: Diabetic Association of Bangladesh
BIRDEM: Bangladesh Institute of Research and Rehabilitation for Diabetes, Endocrine and Metabolic Disorders
GDM: Gestational diabetes mellitus
DM: Diabetes mellitus
NFG: Normal fasting glucose
FPG: Fasting plasma glucose
2hPG: 2 h after 75 g plasma glucose
MADRS: Montgomery-asberg depression rating scale
BMI: Body mass index
WHR: Waist hip ratio
BP: Blood pressure
SBP: Systolic blood pressure
DBP: Diastolic blood pressure
HTN: Hypertension
CI: Confidence interval
OR: Odds ratio
